# Classification of Non-Indigenous Species Based on Their Impacts: Considerations for Application in Marine Management

**DOI:** 10.1371/journal.pbio.1002130

**Published:** 2015-04-15

**Authors:** Henn Ojaveer, Bella S. Galil, Marnie L. Campbell, James T. Carlton, João Canning-Clode, Elizabeth J. Cook, Alisha D. Davidson, Chad L. Hewitt, Anders Jelmert, Agnese Marchini, Cynthia H. McKenzie, Dan Minchin, Anna Occhipinti-Ambrogi, Sergej Olenin, Gregory Ruiz

**Affiliations:** 1 Estonian Marine Institute, University of Tartu, Pärnu, Estonia; 2 National Institute of Oceanography, Israel Oceanographic and Limnological Research, Haifa, Israel; 3 Environmental Research Institute, University of Waikato, Hamilton, New Zealand; 4 Maritime Studies Program of Williams College and Mystic Seaport, Mystic, Connecticut, United States of America; 5 Instituto do Mar, Department of Oceanography and Fisheries, University of the Azores, Horta, Portugal; 6 MARE—Marine and Environmental Sciences Centre, Funchal, Portugal; 7 Marine Invasions Research Laboratory, Smithsonian Environmental Research Center, Edgewater, Maryland, United States of America; 8 Scottish Marine Institute, Scottish Association for Marine Science, Oban, United Kingdom; 9 Department of Biology, Wayne State University, Detroit, Michigan, United States of America; 10 School of Science, University of Waikato, Hamilton, New Zealand; 11 Flødevigen Marine Research Station, Institute of Marine Research, His, Norway; 12 Department of Earth and Environmental Sciences, University of Pavia, Pavia, Italy; 13 Northwest Atlantic Fisheries Centre, Fisheries and Oceans Canada, St. John’s, Newfoundland, Canada; 14 Marine Science and Technology Center, Klaipeda University, Klaipeda, Lithuania; Simon Fraser University, CANADA

## Abstract

Assessment of the ecological and economic/societal impacts of the introduction of non-indigenous species (NIS) is one of the primary focus areas of bioinvasion science in terrestrial and aquatic environments, and is considered essential to management. A classification system of NIS, based on the magnitude of their environmental impacts, was recently proposed to assist management. Here, we consider the potential application of this classification scheme to the marine environment, and offer a complementary framework focussing on value sets in order to explicitly address marine management concerns. Since existing data on marine NIS impacts are scarce and successful marine removals are rare, we propose that management of marine NIS adopt a precautionary approach, which not only would emphasise preventing new incursions through pre-border and at-border controls but also should influence the categorisation of impacts. The study of marine invasion impacts requires urgent attention and significant investment, since we lack the luxury of waiting for the knowledge base to be acquired before the window of opportunity closes for feasible management.

## Abstract 2

Classifying the impact of non-indigenous species presents special problems in marine environments. This Essay presents a framework that focuses on values and emphasizes precaution in managing the data limitations and uncertainties found in the marine context.

## Introduction

Impacts caused by non-indigenous species (NIS), both in ecological and economic/societal terms, are of great importance to legislators, managers, policy makers, and conservationists in terms of prioritising management actions and funding streams. Assessment of such impacts has therefore received substantial attention from scientists recently and is one of the focus areas of bioinvasion science both in terrestrial and aquatic realms [1–4]. A classification system of NIS impacts may provide a valuable tool for management, especially a classification system that considers existing obligations and regulatory frameworks.

Recently, Blackburn and colleagues [5] proposed a classification scheme of alien species based on the magnitude of impact in response to the wide variability observed in impacts across locations, species, and ecosystems. Their intent was to produce a unified framework to address five key aims, including prioritisation of management actions. A companion article [4] is forthright in prioritising impacts for “facilitating the risk assessment and management of alien species”. We note that numerous ecosystem differences exist that differentiate marine from terrestrial systems [6], and there are also differences in terms of knowledge base, isolation, accessibility, and jurisdiction conflicts. It is, therefore, prudent to discuss whether this classification accommodates the realities and challenges inherent in our present state of knowledge of (and relevant data availability for) NIS in the world’s oceans, and to expand upon the considerations for implementing such a scheme.

There are several international legislative instruments and voluntary guidelines targeting evaluation and management of impacts caused by NIS. One of the most demanding international instruments in this regard is the European Union Marine Strategy Framework Directive (MSFD) [7]. The criteria for “good environmental status” according to the MSFD include “impacts of non-indigenous invasive species at the level of species, habitats and ecosystem” [8]. A regulation recently adopted by the European Council on the prevention and management of the introduction and spread of invasive alien species (IAS) [9] defines IAS as those with “a significant negative impact on biodiversity as well as serious economic and social consequences”. A companion European Commission staff working document [10] defines IAS as “alien species whose introduction or spread has been found, through risk assessment, to threaten biodiversity and ecosystem services, or to have a negative impact on the environment, society and the economy”.

Here we present an impact framework that is complementary to that proposed by Blackburn and colleagues [5], focussing exclusively on the marine system and explicitly on management outcomes. In doing so, we highlight specific marine information gaps and outline critical challenges in applying a framework to marine systems. We note that many of the concerns raised in a marine context may be equally applicable to other ecosystems.

## Data Availability Limits the Utility of an Impact Classification System

The concept of indigenous (native) species and their corresponding natural ranges, inter alia, is largely dependent on the scientific knowledge of the actual biota within a given region. An enduring misconception is that because marine biological surveys have been conducted since the 19th century, we have a reasonable measure of confidence in separating NIS from native biota, at least among well-known taxa within many regions. In fact, human-mediated dispersal of species in the ocean preceded such studies by many centuries, and this potentially prejudices our understanding of invasion patterns and processes based on presumptive “baseline” data [11–13] and cryptogenic species (species whose origins are unknown) (Box 2 in [5]).

Moreover, there are few region-wide targeted efforts to survey the presence and abundance of marine NIS, and many such studies are recent (see [14,15]). Due to a lack of study or expertise, discovery of NIS in the invaded area can sometimes lag by decades or longer [16–18]. It is for this reason that the numbers of recorded marine NIS are likely to be grossly underestimated. The magnitude of this gap is difficult to assess, and it varies amongst different taxa, habitats, and regions (e.g., [19]). The datasets are presumably most accurate for large and conspicuous species, but as molecular tools have been increasingly used, they have revealed cryptic species and erroneous identifications even among fish and decapod crustaceans [20–23].

Many marine NIS, particularly smaller sized taxa, including protozoa, bacteria, and viruses, remain unrecognised and undetected because of the continuing erosion of taxonomic expertise [11,24] and lack of priority in surveying NIS (see [14]). Critically, even for those marine NIS that are confirmed, our understanding of their impacts is extremely limited. Impacts for the vast majority of marine NIS remain unknown and have not been quantitatively or experimentally studied over sufficiently long temporal and spatial scales [25], and their cumulative and synergetic connections with other drivers of change affecting the marine environment are largely unknown [26–28]. Entrapped in a catch-22 situation, the number of marine NIS with sufficient data to satisfy the criteria for “significant negative impact” [9] is low, because understanding of marine ecosystem functions is constrained due to lack of appropriately designed studies. Unless impacts are conspicuous, induce direct economic cost, or impinge on human welfare, they fail to arouse public awareness, funding, and scientific analysis.

Unlike in terrestrial and inland aquatic habitats, where bioinvasions impact several types of ecosystem services and affect human welfare [29–31], in marine habitats the majority of demonstrable bioinvasion impacts appear to primarily relate to native biodiversity and ecosystem health. Marine bioinvasion researchers who have studied the impacts of various taxa have invariably commented on the paucity of underlying knowledge [32–35]. This has resulted in a suite of pleas for increased evaluation and cross-system comparison between native and recipient regions as well as between introductions; however, the paucity of data has yet to be rectified [32,36,37].

Only a modest fraction of recorded marine NIS have been formally evaluated for impact. For example, the most recent literature survey on NIS marine macroalgae revealed information on impacts for only twelve species globally, with experimental studies performed with eight species [38]. Katsanevakis and colleagues [39] sorted records of impacts of marine NIS in Europe according to their evidence base (manipulative experiments, direct observations, natural experiments, modelling, non-experiment-based correlations, expert judgment). They concluded that evidence for most of the reported ecosystem impacts is weak, as it is based on expert judgement or dubious correlations; only 13% of the reported impacts were inferred from manipulative or natural experiments. Ruiz and colleagues [32,40] reported a similar paucity of data on impacts for marine NIS in North America. Hewitt and colleagues [41] found that for 657 global marine non-indigenous biofouling species, only 164 (25%) had demonstrable or inferred impacts in at least one core value (environmental, economic, social, or cultural), using consequence matrices. For the remaining 493 species, no information could be found. Schaffelke and Hewitt [42] and Williams and Smith [43] independently found impact information for approximately 6% of known macroalgal invaders. Finally, Zaiko and colleagues [44] found that ecological impacts had been documented for 36% of the known NIS and cryptogenic species in the Baltic Sea.

**Fig 1:**
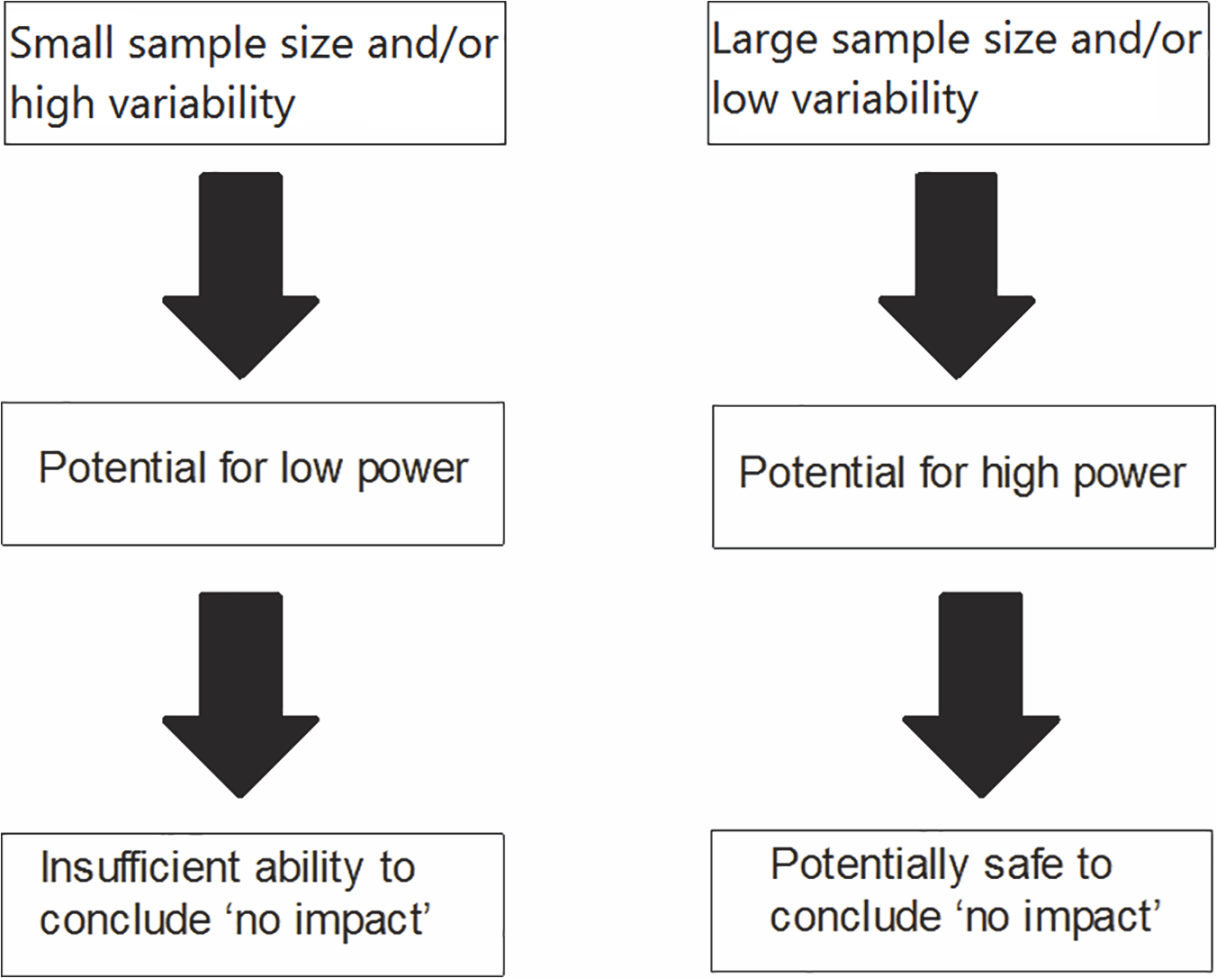
Relation between the sample size and variability of data, the power of a test, and concluding a non-indigenous species has no impact.

The problem of sparse data is exacerbated by a propensity to “miss” impacts far more frequently than to falsely attribute them (Box 1 and Fig 1). Davidson and Hewitt [45] argued that the reliance on significance testing that focusses on preventing type I errors, coupled with the arbitrary and traditionally accepted cut-off for significance at α = 0.05, has led to low power in the majority of NIS studies for detecting any impact. The authors identified the highly restricted suite of studies where a formal manipulative comparison was made but no impact was detected. The majority of these studies (97%) had insufficient power to detect an impact even if it were present. Unfortunately, the consequence of failing to detect an impact has often led management to assume that there is no impact. This false certainty is transferred into the policy domain, where species for which no impact has been detected—even if no study took place—are deemed to have no impact and are therefore categorised incorrectly as harmless. The application of an approach that calls for managing only NIS with demonstrated impact [4,5,46] would result in an increased risk of harmful invasions through reliance on non-precautionary interpretation of research outcomes [2,45,47,48]. Where research on impacts yields insignificant results, these findings should be considered carefully with respect to their role in the risk assessment and management process. Emphasis should be placed on the associated effect size, whether or not this effect was significant at 0.05. In deciding how to then incorporate these impact data, managers should assess the potential power of the study. Where sample sizes are small and/or variability in observations/data is high (i.e., power is likely low), it may be appropriate to incorporate these findings into the compilation of impact data. Conversely, where sample sizes are large and/or variability low (i.e., power is likely high), it may be safe to assume a NIS has no impact (Fig 1).

**Box 1. Glossary of Considerations of Power in Analysing Impact Evaluations**

Type I error: the error of falsely attributing an effect (false rejection of the null hypothesis).

Type II error: the error of missing an effect (false acceptance of the null hypothesis).

Statistical power (of a test): the probability of correctly rejecting the null hypothesis [49]. Low power can occur when sample or effect sizes are small, or variability is high (Fig 1).

Fallacy of the false negative [50]: incorrectly concluding that a statistically non-significant finding (no detected impact) equates to “no impact”. While power analysis is the ideal tool to determine the potential for missing impacts, studies rarely include such an analysis. In such cases, an approximation of power can assist the risk assessment and management process in determining how to treat statistically insignificant results of impact analyses.

## Are Marine Non-Indigenous Species Manageable Based on Impacts?

We believe that the paucity of data on marine NIS presents a special challenge for understanding invasion ecology and prioritising conservation and research aims for marine ecosystems. In our experience, marine NIS that qualify as “data deficient” are excluded from further evaluation [41,51]. But a precautionary approach would treat data-deficient NIS as representing an unknown, and therefore these species should be considered as a source of high risk (though we note this is likely to pose significant challenges).

A NIS classification framework is of practical value only if it can be populated with a sufficient amount of scientifically validated data and then used for management purposes (see Table 1 of [5]) such that management focusses solely on species known to cause harm [4,46]. By adopting this approach, however, we fail to properly manage or allow for the management of the unknowns. Hence, we fail to adequately consider Knightian uncertainty (after [52]), which in this context is strongly linked to a species’s fundamental niche. Such a direction may save management funds, but will inevitably cause more damage (and/or costs) as unanticipated invasions occur.

Limiting evaluation to demonstrable impacts is fundamentally a non-precautionary approach for NIS management. We rarely, if ever, have sufficient knowledge—or the luxury of waiting for such a knowledge base to be acquired—before the window of opportunity closes for feasible management, particularly in the marine environment. Also, we may not know what constitutes the greatest degree of impact. For example, we may not have the ability to demonstrate reversibility through removal; instead, the opportunity to consider heuristic understanding based on expert opinion may be a more practical approach (see [41] and S1 Table).

Removal strategies (including both eradication and control) generally target “pest” or “nuisance” NIS that have demonstrable negative ecological and socio-economic impacts. While removal of several NIS from terrestrial and inland water habitats has proven effective [53], many (if not most) marine attempts have failed, e.g., the veined whelk *Rapana venosa* (Valenciennes, 1846) along the Brittany coast, France [54]; the Japanese kelp *Undaria pinnatifida* (Harvey) Suringar, 1873, in Italy, the United Kingdom, the Netherlands, New Zealand, Australia, and the United States (California) (summarised in [55]); the sea squirt *Didemnum vexillum* (Kott, 2002) in New Zealand [56] and the United Kingdom [57]; and the fanworm polychaete *Sabella spallanzanii* (Gmelin, 1791) in New Zealand [58]. The few successful removals of marine NIS include the black-striped mussel *Mytilopsis sallei* in three lock-gated marinas in Darwin, Australia [59]; the South African sabellid polychaete *Terebrasabella heterouncinata* (Fitzhugh & Rouse 1999) in the vicinity of abalone farms in California [60]; an isolated incursion of *U*. *pinnatifida* in the Chatham Islands, New Zealand [61]; and *Caulerpa taxifolia* (M. Vahl) C. Agardh, 1817, in Agua Hedionda Lagoon and Huntington Harbor, California [62], and in sheltered embayments in New South Wales, Australia [63]. The success of these efforts was due to early discovery, occurrence within confined habitats, limited spatial spread, and a rapid response from management with sufficient funding. While several removal activities have been attempted to control invasive populations in marine environments, none have apparently succeeded in reversing the impact of an invasion for any enduring time [64].

Finally, the precautionary approach is necessary because notable impacts may become clear or problematic only long after the invasion. Assuming that NIS are a priori harmless until proven harmful results in assuming no impact and accounting for impact only after it has been demonstrated. In the case of marine invasions, that would be far too late for any meaningful management action. The true application of precaution for introduced species would require that we assume impact until we cannot support that premise (e.g., [45,51,65,66]). The pervasive bias of the precautionary approach is further exhibited in the approach to hypothesis testing (see [45]). Therefore, management of marine NIS cannot rely solely on evidence on impacts.

## Alternative Impact Evaluation Frameworks for Management

While NIS impact studies are often performed by research scientists and involve fundamental research components, outputs of such studies are of practical relevance for managers. However, appropriate NIS impact evaluation frameworks could have an even higher practical value if they not only considered the availability/uncertainty of data, but also involved multiple types of impact (i.e., environmental, social, economic, cultural) that NIS can have.

Consequence matrices associated with environmental, economic, social, and cultural values that explicitly delineate rankings have been developed for biosecurity purposes to categorise and incorporate impact into a variety of semi-quantitative risk assessments [40,41,67,68]. These matrices represent guidelines to aid in determining the level of possible impact and therefore are based on both evidentiary support and inference, through expert and stakeholder assessment or modelling approaches [44,67–70]. We developed such a matrix for NIS impacts, with the value sets and approach outlined in Box 2 and impact thresholds and exemplars provided in S1 Table. It should be noted that the exemplars are not exhaustive and that not all exemplars are required to assign a level of impact.

To ensure a consistent approach, exemplars of impact were developed to gauge impact against the following: information gaps, local scale of impact (percentage of area, percentage of value), scale/extent of impact (local, national, and/or international), and anticipated resilience (ability to recover).

Threshold values for impacts (S1 Table) were established via evaluation of relevant legislative and policy obligations, and subsequently adjusted based on expert consultations held in New Zealand, Australia, and South America during previous research (e.g. [41,68,69]). Threshold values are based on consensus, and represent a perceived value rather than a fixed value.

The consequences for each value subcategory provide multiple exemplars of varying levels of impact (ranging from negligible to extreme), not all of which are required for that level of impact to be considered relevant.

After inspecting several other impact matrices developed and applied recently (e.g., [5,71,72]) relative to those developed for biosecurity management purposes, we hereby suggest that any impact evaluation framework (for details, see Box 2 and S1 Table) should meet the following criteria.

1. The framework should be focussed on the value sets being protected (see Box 2) rather than on the mechanism of impact (as in [5]). The latter approach is critically flawed as an input to a risk process and is less relevant to the wider considerations of managers and decision makers. *How* a value is impacted is irrelevant from a risk management perspective, given that the risk is consequence multiplied by likelihood. Such an approach has explicitly worked with biosecurity managers and identified value sets of particular relevance to decision making, such as internationally and nationally legislated protections (protected species, habitats), and high value areas of special interest (UNESCO World Heritage Sites, marine protected areas) (see [41,67–70]).
2. The framework should involve assessment of the impact values against background variability, due to the need to support empirical means of detection. However the exemplars in S1 Table should not be rigid requirements, but rather examples of what constitutes a level of impact. The framework could include the concept of the recovery that could be expected if the species was removed, highlighting the heuristic/expert opinion approach we have taken to evaluate impact.
3. The framework should adopt standardised risk assessment terminology (negligible, very low, low, moderate, high, extreme) in describing a progressive increase in the degree of impact within value sets. This categorisation of increasing degree of impact should identify exemplars of increasing spatial and temporal effect on the explicit value set. This categorisation of increasing degree of impact could also incorporate an increase in organisational scale “so that a new level of organisation is involved” [5].
4. The framework should be readily and consistently extended into economic, social, and cultural values and not consider environmental impacts alone (see Box 2 and S1 Table). Given the legislative mandates for the consideration of impacts to human welfare as well as to the environment (e.g., [8,10]), any framework used in risk assessment needs to explicitly describe how impacts to economic, social, and cultural values should also be assessed and to demonstrate a consistency of approach across values (for details, see Box 2 and S1 Table).

**Box 2. Examples of value sets for categorisation (modified from [40,68])**

Consequences (impacts) were assessed against the following four core values and a number of subcategories.

- Environmental: biological and physical characteristics of an ecosystem that may potentially be impacted by NIS, excluding extractive commercial, traditional, or recreational use and aesthetic value. Exemplars are provided for impacts on habitat, biodiversity, trophic interactions, nationally important and ecologically valuable species, and assets of environmental significance (S1 Table).
- Economic: components within an ecosystem that provide a current or potential economic gain or loss, including fisheries resources and commercially relevant infrastructure (e.g., port infrastructure, offshore wind and tidal generation, desalination plants).
- Social and cultural: values placed on a location or species in relation to human use for pleasure, aesthetic, and generational values. This value category also takes into account iconic or spiritual value, including locations that create a sense of local, regional, or national identity. In this report, we have assessed impacts on social, cultural, aesthetic, and national image.
- Human health: value of a safe and healthy society shared equally across generations and socio-economic groups.

Furthermore, any impact evaluation framework should be conceptually based on reliable information about the source and mechanism of invasion. However, a lack of such data should not relegate a data-deficient species to the low impact category but should instead place the species in a high risk/threat category, as we do not know the potential impact(s) (S1 Table). Whether a species is data-deficient is open to interpretation as no clear benchmarks exist for this assessment. We have created a benchmark for assessing data-deficient species through the use of impact categorisations (see consequence matrices in S1 Table). Using benchmarks, such as consequence matrices, data-deficient species are assessed based on currently available knowledge that can be updated when more information becomes available. This ensures that these species are assessed within a risk context instead of being sidelined from the assessment as data-deficient (see discussion in [41]).

The assessment of data-deficient species is important, as the status of only a small fraction of described marine animal species has been evaluated by the International Union for Conservation of Nature [73]. For example, the majority of marine ray-finned fish and marine invertebrates are determined to be “unreviewed” [74]. Explicitly aligning impact categorisation with a risk-based process will provide greater consistency between scientific investigation and understanding of impacts and the biosecurity management actions (including decisions) that ensue.

## Management of Marine Bioinvasions and the Value of Impact Studies: The Reality

Based on the considerations presented above, the practical approach and the only currently viable measure for managing marine NIS is pre-border management (i.e., controlling invasion vectors and pathways to prevent introductions) and at-border management, where arrivals involving targeted species can be directly managed. Once a NIS has been established in a new area, management efforts could be directed at rapid identification of its spatial range and its potential for spread, and communication with managers in regional NIS bridgeheads and hubs to delay, and perhaps control, secondary spread. Should a potentially harmful NIS be discovered in an enclosed habitat, rapid removal may be attempted. Models predicting direction and extent of possible secondary spread [75,76] may advise monitoring actions and support management decisions. In some cases, where the natural dispersal capability of an NIS exceeds any management attempt to control its spread, a mitigation approach may be the only appropriate response [15].

Thus, the value and importance of NIS impact assessments in marine ecosystems may differ from those in terrestrial and inland water ecosystems, where impacts are better understood and removal may be feasible. Impact assessments are indeed important in a wider marine ecosystem/fisheries management context to adapt management regimes to new situations caused by NIS invasions, but these assessments are generally of less use in practical management of NIS, given the near impossibility of removal. However, given the strict context dependence, impact assessments might be of high value in managing specific NIS in particular localities (such as harmful algal blooms [77]). We suggest marine bioinvasions management should be based on the precautionary principle and should primarily focus on invasion pathway/vector management to minimise the risks of new introductions, with knowledge on impact coupled with likelihood of invasion to inform and support pre-border management decisions.

Of practical concern is that management often accepts on face value an assessment that a NIS has no impact, and is unlikely to re-evaluate that status, unless the species’s impact proves extreme, has severe health or economic implications, or becomes a public concern. Management decisions are made on the best available data at the time, and the downstream flow of these decisions is often difficult to reverse, despite declarations of “adaptive management”. For example, allowing the importation of an aquaculture species or the harvest of an introduced species is difficult to reverse. Stakeholders, having been led to believe that the risks are nonexistent or low, develop and invest in businesses that use these NIS with government approval (e.g., [78]). Revoking these rights because of a re-assessment of impact has a number of legal, social, and economic repercussions for management. In addition, management often has limited time and resources; having the opportunity initially to make a fully informed decision is often difficult, let alone having a second opportunity to re-evaluate. As a consequence, the political will to devote resources to revising an initial impact assessment that indicated a nonexistent or low level of risk will rarely exist.

## Conclusions

The dearth of NIS impact studies in marine ecosystems and associated information on socio-economic consequences poses major challenges for managers trying to address bioinvasions in a comprehensive manner. We recognise that characterising marine invasion impacts requires urgent attention and intensified efforts to fully understand the real impact of one of the most important human-induced stressors affecting marine ecosystems—bioinvasions. Ultimately, impact data may facilitate informed response frameworks, critical to understanding and assigning risk, that can inform management. Given that impact data and post-invasion risk assessments are scarce, the application of the precautionary principle must remain in force, with management focussing on prevention of new incursions through management of invasion vectors and pathways.

## Supporting Information

S1 Table Exemplars of impact within different value sets in the proposed consequence scheme (Box 2). Material derived from [41].
(DOCX)

## Abbreviations

IAS: invasive alien species
MSFD: Marine Strategy Framework Directive
NIS: non-indigenous species
