# Supplementary material for: Classification of Non-Indigenous Species Based on Their Impacts: Considerations for Application in Marine Management
Source: PLoS Biol. 2015 Apr 15;13(4):e1002130. doi: 10.1371/journal.pbio.1002130 (PMC4398364; doi:10.1371/journal.pbio.1002130)
Supplement: S1 Table — Material derived from [41]. (DOCX) [file pbio.1002130.s001.docx]

**S1 Table.** Exemplars of impact to different value sets in the proposed consequence scheme (Box 2). Material derived from [41].

| Value |  | Negligible to Very Low | Low | Moderate | High | Extreme |
| --- | --- | --- | --- | --- | --- | --- |
| Habitat and habitat forming species | Information Gap |  |  |  | • Limited information is available on the identity and distribution of habitat types; limited information is available on the identity of habitat-forming species and their susceptibility to the NIS |  |
|  | Local area of value impacted | • No significant changes to habitat types observed; | • Localised affects on habitat in <10% of total habitat area; | • <30% of habitat area affected/replaced; | • <70% of habitat area affected/replaced; | • >70% of habitat area affected/replaced; |
|  | Alteration of value | • no new habitat type observed in the invaded area; | • measurable changes to habitat types, new habitat type observed; | • moderate changes to habitat types, new habitat type(s) observed, possible loss of at least one habitat type; | • major changes to habitat types, new habitat types observed, loss of >30% pre-existing habitat types; | • significant changes to habitat types, few pre-existing habitat types existing (>70% loss); |
|  |  | • populations of habitat forming species are not affected (<1% change); | • <10% reduction in population abundances of habitat forming species | • <30% reduction in population abundances of habitat forming species | • <70% reduction in population abundances of habitat forming species; | • >70% reduction in population abundances of habitat forming species; |
|  |  |  |  |  | • local/ecological extinction of at least one habitat forming species | • local/ecological extinction of more than one habitat forming species;  global extinction of one habitat forming species |
|  | Spatial scale | • Impacts affecting <1% of area of any habitat type | • Impacts occurring at local scales | • Impacts occurring at a local to national region scale | • Impacts occurring at a national scale | • Impacts occurring at international region scales |
|  | Temporal reversibility | • Based on expert opinion, if the NIS could be removed, recovery would be expected in days; changes in habitat not measurable against background variability. | • Based on expert opinion, if the NIS could be removed, recovery would be expected in days to a year; no loss of habitat-forming species populations | • Based on expert opinion, if the NIS could be removed, recovery would be expected in less than a decade; no loss of habitat-forming species | • Based on expert opinion, if the NIS could be removed, recovery would be expected in centuries; loss of habitat types and habitat-forming species; local extinction events | • Based on expert opinion, even if the NIS could be removed, recovery would not be expected; loss of multiple habitat types and habitat forming species populations causing significant local extinction; global extinction of at least one species |

| Value |  | Negligible to Very Low | Low | Moderate | High | Extreme |
| --- | --- | --- | --- | --- | --- | --- |
| Biodiversity | Information Gap |  |  |  | • Limited information is available on the distribution of the biodiversity relative to the NIS distribution; limited information is available on the susceptibility to the NIS or the vulnerability of life history stages of these species |  |
|  | Local area of value impacted | • No change in species richness or abundance in presence of NIS detetcted relative to background vairability | • Species richness or abundance is detectably reduced in NIS presence in a small area compared to known areas of distribution (<20%) | • Species richness or abundance is detectably reduced in NIS presence in a moderate area compared to known areas of distribution (<40%) | • Species richness or abundance is detectably reduced in NIS presence in a large area compared to known areas of distribution (<70%); | • Species richness or abundance is detectably reduced in NIS presence in a moderate area compared to known areas of distribution (>70%); |
|  | Alteration of value | • Biodiversity impact by the NIS is not differentiable relative to background variability | • 20% Species richness or abundance is detectably reduced in NIS presence | • 40% Species richness or abundance is detectably reduced in NIS presence | • 70% Species richness or abundance is detectably reduced in NIS presence | • >70% Species richness or abundance is detectably reduced in NIS presence |
|  |  | • Reductions in species richness and composition are not readily detectable (<10% variation) | • Reductions in species richness and composition are <20% | • Reductions in species richness and composition are <30% | • Reductions in species richness and composition are <70% | • Reductions in species richness and composition are >70% |
|  |  |  |  |  | • local/ecological extinction of at least one species | • local/ecological extinction of more than one species;  global extinction of one species |
|  | Spatial scale | • Impacts occurring at a local scale | • Impacts occurring at a local scale | • Impacts occurring at a national region scale | • Impacts occurring at a national scale | • Impacts occurring at international region scales |
|  | Temporal reversibility | • Based on expert opinion, if the NIS could be removed, recovery would be expected in days; no change in species richness or composition. | • Based on expert opinion, if the NIS could be removed, recovery would be expected in days to months; no loss of species populations; no local extinctions | • Based on expert opinion, if the NIS could be removed, recovery would be expected in years to decades; loss of at least one species or populations; local extinction events. | • Based on expert opinion, if the NIS could be removed, recovery would be expected in centuries; loss several species or populations; multiple local extinction events; one regional extinction | • Based on expert opinion, even if the NIS could be removed, recovery would not be expected; loss of multiple species of populations causing significant local extinctions; global extinction of at least one species |

| Value |  | Negligible to Very Low | Low | Moderate | High | Extreme |
| --- | --- | --- | --- | --- | --- | --- |
| Trophic interactions (ecosystem) | Information Gap |  |  |  | • Limited information is available on the species composition and abundances of trophic levels and their susceptibility to the NIS; limited information is available on the trophic interactions and fundamental ecosystem processes |  |
|  | Local area of value impacted | NA | NA | NA | NA | NA |
|  | Alteration of value | • No significant changes trophic level species composition observed; no change in relative abundance of trophic levels (based on biomass) | • Minor changes (<10%) in relative abundance of trophic levels (based on biomass); | • Measurable changes (<30%) in relative abundance of trophic levels (based on biomass); | • Major changes (<70%) in relative abundance of trophic levels (based on biomass); | • >70% change in relative abundance of trophic levels (based on biomass); |
|  |  |  | • <10% reduction of population abundances for top predator species | • <30% reduction of population abundances for top predator species | • <70% reduction of population abundances for top predator species; | • >70% reduction of population abundances for top predator species; |
|  |  |  |  |  | • <30% reduction of population abundances for primary producer species | • >30% reduction of population abundances for primary producer species |
|  | Spatial scale |  | • Impacts occurring at local scales | • Impacts occurring at national region scales | • Impacts occurring at national scales | • Impacts occurring at international region scales |
|  | Temporal reversibility | • Based on expert opinion, if the NIS could be removed, recovery would be expected in days to weeks; changes in trophic interactions not measurable against background variability. | • Based on expert opinion, if the NIS could be removed, recovery would be expected in weeks to months; no loss of keystone species populations | • Based on expert opinion, if the NIS could be removed, recovery would be expected in years to decades; loss of keystone species populations; no loss of primary producer populations | • Based on expert opinion, if the NIS could be removed, recovery would be expected in centuries; loss of keystone species populations; changes in trophic levels; loss of primary producer populations; local extinction events. | • Based on expert opinion, even if the NIS could be removed, recovery would not be expected; loss of trophic levels; potential trophic cascades resulting in significant changes to ecosystem structure, alteration of biodiversity patterns and changes to ecosystem function; significant local extinctions |

| Value |  | Negligible to Very Low | Low | Moderate | High | Extreme |
| --- | --- | --- | --- | --- | --- | --- |
| Nationally important and ecologically valuable species | Information Gap |  |  |  | • Limited information is available on the susceptibility to impact or the behavioural vulnerability of the nationally important and/or ecologically valuable species to the NIS |  |
|  | Local area of value impacted | • No nationally important and/or ecologically valuable species impacted by NIS; impacts on behaviour not detectable | • NIS impact to Nationally important and/or ecologically valuable species is restricted to <1% of compared to Nationally important and/or ecologically valuable species' ranges | • NIS impact to Nationally important and/or ecologically valuable species is restricted to <10% of compared to Nationally important and/or ecologically valuable species' ranges | • NIS impact to Nationally important and/or ecologically valuable species is restricted to <20% of compared to Nationally important and/or ecologically valuable species' ranges | • NIS impact to Nationally important and/or ecologically valuable species is deetcted in >20% of compared to Nationally important and/or ecologically valuable species' ranges |
|  | Alteration of value | • No nationally important and/or ecologically valuable species impacted by NIS; impacts on behaviour not detectable | • The number of Nationally important and/or ecologically valuable species impacted by NIS is <1% compared to impact from other hazards | • The number of Nationally important and/or ecologically valuable species impacted by NIS is <10% compared to impact from other hazards | • The number of Nationally important and/or ecologically valuable species impacted by NIS is <20% compared to impact from other hazards | • The number of Nationally important and/or ecologically valuable species impacted by NIS is >20% compared to impact from other hazards |
|  |  |  | • Reductions in nationally important and/or ecologically valuable species population abundances are <1% | • Reductions in nationally important and/or ecologically valuable species population abundances are <10% | • Reductions in nationally important and/or ecologically valuable species population abundances are <20% | • Reductions in nationally important and/or ecologically valuable species population abundances are >20% |
|  | Spatial scale | NA | NA | NA | NA | NA |
|  | Temporal reversibility | • Based on expert opinion, if the NIS could be removed, recovery would be expected; no loss of nationally important and/or ecologically valuable individuals. | • Based on expert opinion, if the NIS could be removed, recovery would be expected in months to years; no loss of nationally important and/or ecologically valuable species populations | • Based on expert opinion, if the NIS could be removed, recovery would be expected in years to decades; no loss of nationally important and/or ecologically valuable species populations; potential loss of genetic diversity. | • Based on expert opinion, if the NIS could be removed, recovery would be expected in centuries; loss of nationally important and/or ecologically valuable species populations causing local extinction; measurable loss of genetic diversity | • Based on expert opinion, even if the NIS could be removed, recovery would not be expected; loss of nationally important and/or ecologically valuable species populations causing global extinction; local extinction of multiple nationally important and/or ecologically valuable species; significant loss of genetic diversity of multiple nationally important and/or ecologically valuable species |

| Value |  | Negligible to Very Low | Low | Moderate | High | Extreme |
| --- | --- | --- | --- | --- | --- | --- |
| Assets (places) of environmental significance | Information Gap |  |  |  | • Limited information is available on the assets of environmental significance and their susceptibility to the NIS |  |
|  | Local area of value impacted | • No significant changes to assets of environmental significance | • Localised ( <10% of total asset area) effects on assets of environmental significance | • Regional (<30% of total asset area) effects on assets of environmental significance | • Regional (<70% of total asset area) effects on assetss of environmental significance; | • National (>70% of total asset area) effects on assets of environmental significance affected/removed; significant changes to assets of environmental significance |
|  | Alteration of value | • No significant changes to assets of environmental significance | • <10% reduction in intrinsic value of at least one asset of environmental significance | • <30% reduction in intrinsic value ofat least one asset of environmental significance | • <70% reduction in intrinsic value ofat least one asset of environmental significance | • >70% reduction in intrinsic value of at least one asset of environmental significance |
|  |  |  |  | • More than one asset of environmental significance affected/removed | • More than 30% of assets of environmental significance affected/removed | • More than 50% of assets of environmental significance affected/removed |
|  |  |  |  | • localised loss of at least one asset of environmental significance | • Regional loss of at least one asset of environmental significance | • National/Global loss of more than one asset of environmental significance |
|  | Spatial scale | NA | NA | NA | NA | NA |
|  | Temporal reversibility | • Based on expert opinion, if the NIS could be removed, recovery would be expected in days; changes in assets of environmental significance not measurable against background variability. | • Based on expert opinion, if the NIS could be removed, recovery would be expected in weeks to months; no complete loss of assets of environmental significance | • Based on expert opinion, if the NIS could be removed, recovery would be expected in years to decades; no complete loss of assets of environmental significance | • Based on expert opinion, if the NIS could be removed, recovery would be expected in centuries; minimal loss of assets of environmental significance | • Based on expert opinion, even if the NIS could be removed, recovery would not be expected; national/global loss of multiple assets of environmental significance |

| Value |  | Negligible to Very Low | Low | Moderate | High | Extreme |
| --- | --- | --- | --- | --- | --- | --- |
| Economic values | Information Gap |  |  |  | • Limited information is available on economic impacts of the NIS |  |
|  | Local area of value impacted | • No discernable change in area of economic activities | • Economic activity is reduced to 99% of its original area (spatial context) | • Economic activity is reduced to less than 95% of its original area (spatial context) | • Economic activity is reduced to less than 90% of its original area (spatial context) | • Economic activity is reduced to less than 90% of its original area (spatial context) |
|  | Alteration of value | • Reduction in national income (including access to international markets and/or trade) from NIS impact shows no discernible change | • Reduction in national income (including access to international markets and/or trade) from NIS impact is <1% | • Reduction in national income (including access to international markets and/or trade) from NIS impact is 1‐5% | • Reduction in national income (including access to international markets and/or trade) from NIS impact is 5‐10% | • Reduction in national income (including access to international markets and/or trade) from NIS impact is >10% |
|  |  | • Reduction in regional income from NIS impact shows no discernible change | • Reduction in regional income from NIS impact is <30% | • Reduction in regional income from NIS impact is 30-50% | • Reduction in regional income from NIS impact is 50-70% | • Reduction in regional income from NIS impact is >70% |
|  |  | • No discernable change in strength of economic activities | • Reduction of strength in individual economic activities is <1% | • Reduction of strength in individual economic activities is 1‐5% | • Reduction of strength in individual economic activities is 5‐10% | • Reduction of strength in individual economic activities is >10% |
|  |  | • No discernable damage or deterioration of infrastructure used by a significant proportion of people (>80% of local population) | • <10% damage or deterioration of infrastructure used by a significant proportion of people (>80% of local population) across a local area | • <30% or deterioration of infrastructure used by a significant proportion of people (>80% of local population) across a national region scale | • <70% damage or deterioration of infrastructure used by a significant proportion of people (>80% of local population) across a nation | • >70% damage or deterioration of infrastructure used by a significant proportion of people (>80% of local population) across international region |
|  | Spatial scale |  | • Impacts occurring at local scales | • Impacts occurring at a local to national region scale | • Impacts occurring at a national scale, influencing markets | • Impacts occurring at international region scales influencing global markets |
|  | Temporal reversibility | • Based on expert opinion, if the NIS could be removed, recovery would be expected in days. | • Based on expert opinion, if the NIS could be removed, recovery would be expected in weeks to months, no loss of any economic industry. | • Based on expert opinion, if the NIS could be removed, recovery would be expected in less than a year with the loss of at least one economic activity. | • Based on expert opinion, if the NIS could be removed, recovery would be expected in less than 5 years with the loss of at least one economic activity. | • Based on expert opinion, even if the NIS could be removed, recovery would not be expected in less than 10 years with the loss of multiple economic activities. |

| Value |  | Negligible to Very Low | Low | Moderate | High | Extreme |
| --- | --- | --- | --- | --- | --- | --- |
| Social values | Information Gap |  |  |  | • Limited information is available on social impacts of the NIS |  |
|  | Local area of value impacted |  | • Social activity is reduced to less than 90% of its original area (spatial context) within the region | • Social activity is reduced to less than 80% of its original area (spatial context) within the region | • Social activity is reduced to less than 70% of its original area (spatial context) within the region | • Social activity is reduced to less than 60% of its original area (spatial context) within the region |
|  | Alteration of value | • Social activity reduction is minimal (<1%) | • Social activity reduction is <10% | • Social activity reduction is <20% | • Social activity reduction is <40% | • Social activity reduction is >40% |
|  |  | • Degradation of amenity used by 80% of people over an local scale is minimal (<1%) | • <10% degradation of amenity used by 80% of people across a local scale | • <30% degradation of amenity used by 80% of people across a regional scale | • <70% degradation of amenity used by 80% of people across a nation | • >70% degradation of amenity used by 80% of people across a nation, or across international borders |
|  |  | • No significant changes to nationally important places | • Localised affects on nationally important places in <10% of nationally important places; measurable changes to nationally important places; <10% reduction in intrinsic value of nationally important places | • <30% of nationally important places affected; moderate changes to nationally important places; <30% reduction in intrinsic value of nationally important places | • <70% of nationally important places affected; major changes to nationally important places; <70% reduction in intrinsic value of the nationally important places; loss of at least one nationally important place | • >70% of nationally important places affected; significant changes to nationally important places; loss of more than one nationally important place |
|  |  | • No discernable change in strength of social activities | • Reduction of strength in separate social activities is <10% | • Reduction of strength in separate social activities is <20% | • Reduction of strength in separate social activities is <40% | • Reduction of strength in separate social activities is >40% |
|  | Spatial scale |  | • Social activity reduction is restricted to the locality of incursion/impact | • Social activity reduction is restricted to the country region of incursion/impact | • Social activity is reduced at national scales | • Social activity is reduced in international regions |
|  | Temporal reversibility | • Based on expert opinion, if the NIS could be removed, recovery would be expected in days. | • Based on expert opinion, if the NIS could be removed, recovery would be expected in weeks to months, no loss of any social activities. | • Based on expert opinion, if the NIS could be removed, recovery would be expected in less than a year and loss of at least one tourism activities. | • Based on expert opinion, if the NIS could be removed, recovery would be expected in less than a decade and loss of at least one tourism activities. | • Based on expert opinion, if the NIS could be removed, recovery would not be expected and loss of multiple tourism activities. |

| Value |  | Negligible to Very Low | Low | Moderate | High | Extreme |
| --- | --- | --- | --- | --- | --- | --- |
| Cultural values | Information Gap |  |  |  | • Limited information is available on cultural impacts of the NIS |  |
|  | Local area of value impacted | • No significant changes to culturally important places | • Localised affects on culturally important places in <10% of culturally important places; measurable changes to culturally important places; <10% reduction in intrinsic value of culturally important places | • <30% of culturally important places affected; moderate changes to nationally important places; <30% reduction in intrinsic value of culturally important places | • <70% of culturally important places affected; major changes to culturally important places; <70% reduction in intrinsic value of the culturally important places; loss of at least one culturally important place | • >70% of culturally important places affected; significant changes to culturally important places; loss of more than one culturally important place |
|  | Alteration of value | • Cultural activity reduction is minimal (<1%) | • Cultural activity reduction is <10% | • Cultural activity reduction is <20% | • Cultural activity reduction is <40% | • Cultural activity reduction is >40% |
|  |  | • Degradation of cultural amenities used by 80% of people over an local scale is minimal (<1%) | • <10% degradation of cultural amenities used by 80% of people across a local scale | • <30% degradation of cultural amenities used by 80% of people across a regional scale | • <70% degradation of cultural amenities used by 80% of people across a nation | • >70% degradation of cultural amenities used by 80% of people across a nation, or across international borders |
|  |  | • No discernable change in strength of cultural activities | • Reduction of strength in separate cultural activities is <10% | • Reduction of strength in separate cultural activities is <20% | • Reduction of strength in separate cultural activities is <40% | • Reduction of strength in separate cultural activities is >40% |
|  |  |  | • Cultural activity is reduced to less than 90% of its original area (spatial context) within the region | • Cultural activity is reduced to less than 80% of its original area (spatial context) within the region | • Cultural activity is reduced to less than 70% of its original area (spatial context) within the region | • Cultural activity is reduced to less than 60% of its original area (spatial context) within the region |
|  | Spatial scale |  | • Cultural activity reduction is restricted to the locality of incursion/impact | • Cultural activity reduction is restricted to the country region of incursion/impact | • Cultural activity is reduced at national scales | • Cultural activity is reduced in international regions |
|  | Temporal reversibility | • Based on expert opinion, if the NIS could be removed, recovery would be expected in days. | • Based on expert opinion, if the NIS could be removed, recovery would be expected in weeks to months, no loss of any social activities. | • Based on expert opinion, if the NIS could be removed, recovery would be expected in less than a year and loss of at least one social activities. | • Based on expert opinion, if the NIS could be removed, recovery would be expected in less than a decade and loss of at least one social activity. | • Based on expert opinion, even if the NIS could be removed, recovery would not be expected and loss of multiple social activities. |

| Value |  | Negligible to Very Low | Low | Moderate | High | Extreme |
| --- | --- | --- | --- | --- | --- | --- |
| National image (iconic places or species) | Information Gap |  |  |  | • Limited information is available on the NIS impacts on National Image perceptions |  |
|  | Local area of value impacted | • No significant changes to nationally important places or iconic species | • Localised affects in <10% of nationally important places; iconic species only affected in <10% of range | • <30% of nationally important places affected; iconic species affected in <30% of range | • <70% of nationally important places affected; iconic species affected in <50% of range | • >70% of nationally important places affected; iconic species affected at national scales |
|  | Alteration of value | • National image reduction is minimal (<1%) | • National image intrinsic value reduction is <10% | • National image intrinsic value reduction is <20% | • National image intrinsic value reduction is <40% | • National image intrinsic value reduction is >40% |
|  |  | • No discernable change in strength of national image | • Reduction of strength in separate national image activities is <10% | • Reduction of strength in separate national image activities is <20% | • Reduction of strength in separate national image activities is <40% | • Reduction of strength in separate national image activities is >40% |
|  | Spatial scale |  | • National image reduction is restricted to the locality of incursion/impact | • National image reduction is restricted to the country region of incursion/impact | • National image is reduced at national scales | • National image affects neighbouring countries |
|  | Temporal reversibility | • Based on expert opinion, if the NIS could be removed, recovery would be expected in days. | • Based on expert opinion, if the NIS could be removed, recovery would be expected in weeks to months | • Based on expert opinion, if the NIS could be removed, recovery would be expected in less than a year | • Based on expert opinion, if the NIS could be removed, recovery would be expected in less than a decade; loss of at least one nationally important image | • Based on expert opinion, if the NIS could be removed, recovery would not be expected; loss of more than one nationally important image |

| Value |  | Negligible to Very Low | Low | Moderate | High | Extreme |
| --- | --- | --- | --- | --- | --- | --- |
| Aesthetic values | Information Gap |  |  |  | • Limited information is available on aesthetic impacts of the NIS |  |
|  | Local area of value impacted |  | • Aesthetic appeal is reduced to <90% of its original area (spatial context) within the region of incursion/impact | • Aesthetic appeal is reduced to <60% of its original area (spatial context) within the region of incursion/impact | • Aesthetic appeal is reduced to <30% of its original area (spatial context) within the region of incursion/impact | • Aesthetic appeal is reduced to <10% of its original area (spatial context) within the region of incursion/impact |
|  | Alteration of value | • Aesthetic appeal reduction is minimal (<1%) | • Aesthetic appeal of locations is <10% reduced | • Aesthetic appeal of locations is <20% reduced | • Aesthetic appeal of locations is <40% reduced | • Aesthetic appeal of locations is >40% reduced |
|  |  |  |  | • Reduction of strength in separate aesthetic appeal is <20% | • Reduction of strength in separate aesthetic appeal is <40% | • Reduction of strength in separate aesthetic appeal is >40% |
|  | Spatial scale | • No discernable change in original area of aesthetic appeal (spatial context) within the region | • Aesthetic appeal is reduced in the locality of incursion/impact | • Aesthetic appeal is reduced in country region scales | • Aesthetic appeal is reduced at national scale | • Aesthetic appeal is reduced in international regions |
|  | Temporal reversibility | • Based on expert opinion, if the NIS could be removed, recovery would be expected in days. | • Based on expert opinion, if the NIS could be removed, recovery would be expected in weeks to months | • Based on expert opinion, if the NIS could be removed, recovery would be expected in less than a year | • Based on expert opinion, if the NIS could be removed, recovery would be expected in less than a decade | • Based on expert opinion, even if the NIS could be removed, recovery would not be expected |

| Value |  | Negligible to Very Low | Low | Moderate | High | Extreme |
| --- | --- | --- | --- | --- | --- | --- |
| Human health | Information Gap |  |  |  | • Limited information is available on human health impacts of the NIS |  |
|  | Local area of value impacted | • No discernible decrease in quality of life | • <10% decreases in quality of life in the locality of incursion/impact | • <30% decrease in quality of life in the locality of incursion/impact; 10% decrease in quality of life at country region scales | • <70% decrease in quality of life in the locality of incursion/impact; <30% decrease in quality of life at country region scales; 10% decrease in quality of life at national scales | • >70% decrease in quality of life in the locality of incursion/impact; <30% decrease in quality of life at national scales; 10% decrease in quality of life at international scales |
|  | Alteration of value | • No discernible impact on human health (nuisance level) | • Impact on human health (nuisance level) is <10% in a localised area | • Impact on human health (nuisance level) is <30% in a localised area | • Impact on human health (nuisance level) is <70% in a national area | • Impact on human health (nuisance level) >70% in a national and international area |
|  |  | • No discernible increase in human morbidity | • <10% impact on human morbidity in a localised area | • <30% impact on human morbidity in a localised area | • <70% impact on human morbidity in a national area | • >70% impact on human morbidity in a national and international area |
|  |  | • No discernible increase in human mortality | • No discernible increase in human mortality | • No discernible increase in human mortality | • Localised increase in human mortality (<1%) | • National increase in human mortality (<1%) |
|  | Spatial scale | • No discernible NIS effect on human health | • localised NIS effect on human health | • Country region NIS effect on human health | • National NIS effect on human health | • International region NIS effect on human health |
|  | Temporal reversibility | • Based on expert opinion, if the NIS could be removed, recovery would be expected in days. | • Based on expert opinion, if the NIS could be removed, recovery would be expected in weeks to months, no loss of any human life. | • Based on expert opinion, if the NIS could be removed, recovery would be expected in less than a year, no loss of any human life. | • Based on expert opinion, if the NIS could be removed, recovery would be expected in less than a decade and loss of at least one human life at a national level. | • Based on expert opinion, if the NIS could be removed, recovery would not be expected and loss of multiple human lives across international borders. |
